# On the accuracy of the epigenetic copy machine: comprehensive specificity analysis of the DNMT1 DNA methyltransferase

**DOI:** 10.1093/nar/gkad465

**Published:** 2023-05-29

**Authors:** Sabrina Adam, Viviane Klingel, Nicole E Radde, Pavel Bashtrykov, Albert Jeltsch

**Affiliations:** Institute of Biochemistry and Technical Biochemistry, Department of Biochemistry, University of Stuttgart, Allmandring 31, 70569 Stuttgart, Germany; Institute for Systems Theory and Automatic Control, University of Stuttgart, Pfaffenwaldring 9, 70569 Stuttgart, Germany; Institute for Systems Theory and Automatic Control, University of Stuttgart, Pfaffenwaldring 9, 70569 Stuttgart, Germany; Institute of Biochemistry and Technical Biochemistry, Department of Biochemistry, University of Stuttgart, Allmandring 31, 70569 Stuttgart, Germany; Institute of Biochemistry and Technical Biochemistry, Department of Biochemistry, University of Stuttgart, Allmandring 31, 70569 Stuttgart, Germany

## Abstract

The specificity of DNMT1 for hemimethylated DNA is a central feature for the inheritance of DNA methylation. We investigated this property in competitive methylation kinetics using hemimethylated (HM), hemihydroxymethylated (OH) and unmethylated (UM) substrates with single CpG sites in a randomized sequence context. DNMT1 shows a strong flanking sequence dependent HM/UM specificity of 80-fold on average, which is slightly enhanced on long hemimethylated DNA substrates. To explain this strong effect of a single methyl group, we propose a novel model in which the presence of the 5mC methyl group changes the conformation of the DNMT1-DNA complex into an active conformation by steric repulsion. The HM/OH preference is flanking sequence dependent and on average only 13-fold, indicating that passive DNA demethylation by 5hmC generation is not efficient in many flanking contexts. The CXXC domain of DNMT1 has a moderate flanking sequence dependent contribution to HM/UM specificity during DNA association to DNMT1, but not if DNMT1 methylates long DNA molecules in processive methylation mode. Comparison of genomic methylation patterns from mouse ES cell lines with various deletions of DNMTs and TETs with our data revealed that the UM specificity profile is most related to cellular methylation patterns, indicating that *de novo* methylation activity of DNMT1 shapes the DNA methylome in these cells.

## Graphical Abstract

**Graphical Abstract:**
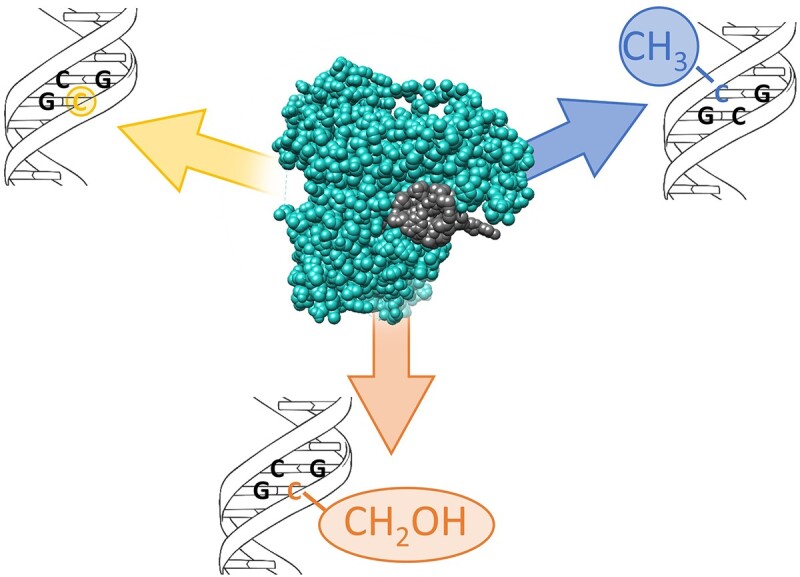

## INTRODUCTION

In multicellular organisms, cell fate and cellular phenotypes are determined by epigenetic mechanisms that are heritable through cell divisions and function without changing the DNA sequence (1). DNA methylation is a key epigenetic process (2–4) which is conserved in most higher eukaryotes (5) and has essential roles in mammalian development and human disease (6,7). In mammals, DNA methylation mainly occurs at the C5 position of cytosine residues, primarily in CpG dinucleotides where most often both DNA strands are methylated in a symmetrical manner (2,8). However, only certain CpG sites are methylated, resulting in the generation of a tissue and cell type-specific methylation pattern which contains epigenetic information. There are 56 million CpG sites in the diploid human genome about 60–80% of which are methylated, corresponding to 4–6% of all cytosines. Methylation levels and patterns vary with cell types and even between alleles, with the largest deviations seen in embryonic stem cells (6,9). 5-methylcytosine can be oxidized to 5-hydroxymethylcytosine, 5-formylcytosine and 5-carboxycytosine by dioxygenases of the TET family (10). While 5-hydroxymethylcytosine may also play signaling roles, 5-formylcytosine and 5-carboxycytosine are excised from DNA by Thymine-DNA glycosylase which initiates active DNA demethylation (11).

In the classical maintenance DNA methylation model (2,4), DNA replication converts the pattern of fully methylated and unmethylated CpG sites into a pattern of hemimethylated and unmethylated sites. Then, DNMT1 specifically methylates the hemimethylated sites, thereby reconstituting the original methylation pattern. To fulfill this important role, DNMT1 has an in-build preference for the methylation of hemimethylated CpG sites (CG/5mCG, HM) over unmethylated sites (CG/CG, UM) that is now called HM/UM specificity. However, this property is incompletely characterized and different studies revealed a wide range of HM/UM specificities with relative rates of CG/CG (UM) substrate methylation of < 1% (12–14), 1–5% (15–17) or even 5–15% (18–20).

Moreover, it is widely discussed that reduced activity of DNMT1 on substrates with hemihydroxymethylated CpG sites (CG/5hmCG, OH) could have an important role in passive DNA demethylation (10,21,22). However, published data of DNMT1 activity on OH substrates varied between <5% (14,23), 5–10% (24) and 20–30% activity (18,25) when compared with HM substrates. Hence, the actual level of the HM/OH specificity, a critical parameter underlying this concept of passive DNA demethylation, is not settled.

This apparent lack of precise information about the relative activity of DNMT1 on UM and OH substrates results from different critical technical limitations which are related to four key aspects.

- First, the flanking sequence dependence of the specificity of DNMT1 has not been considered in most studies. The activity of DNMT1 has been shown to change strongly depending on the flanking sequence of the CpG site (26). However, it has not been studied if and to which extent flanking sequences affect the HM/UM and HM/OH specificity of DNMT1, because all biochemical studies mentioned above were based on only few CpG sites.
- Second, many of the studies listed above provided only a very sparse resolution of the reaction progress curve. This is important, because ratios of fast and slow methylation rates can only be determined accurately, if the reaction progress is sufficiently sampled by data points. At early phases of the reaction, relative rates of disfavored substrates cannot be determined accurately, because of too little turnover. Conversely, at late time points, favored substrates are almost completely turned over, again precluding the determination of accurate reaction rates.
- Third, many studies were conducted using one substrate after the other. However, this is an unrealistic setting, as it does not allow the enzyme to choose between substrates, which may lead to an artificial boost of activity at unfavored substrates. Moreover, in cells methylated and unmethylated CpG sites occur on one continuous DNA molecule, where DNMT1 can slide along and choose the most preferred targets. Still, only very few studies used long hemimethylated DNA substrates to study the specificity of DNMTs.
- Forth, important mechanistic features regarding the specificity of DNMT1 were not resolved. The CXXC domain of the enzyme, which binds to unmethylated DNA, was shown in one study to increase the HM/UM specificity, because a DNMT1 mutant without the CXXC domain showed lower values (27). This effect could be mediated by an auto-inhibitory loop located between the CXXC and the BAH1 domains that was shown to inhibit DNMT1 after DNA binding to the CXXC domain (27). In contrast, another study investigated DNA methylation by a DNMT1 mutant in which DNA binding of the CXXC domain was inactivated, but it did not detect changes of the HM/UM specificity (19).

Here, we investigated the specificity of DNMT1 using a recently developed Deep Enzymology approach (28). In these experiments, libraries of single CpG site substrates with different sequences and modification states are mixed, methylated by DNMT1, a hairpin linker is ligated and the DNA is bisulfite converted. This is followed by next generation sequencing to determine the sequence of individual product molecules together with their methylation state. Based on the sequence and methylation information of very many individual product molecules, the activity of DNMT1 on substrates with different flanking sequences and modification states can be determined. Experiments were conducted using unmethylated (UM), hemihydroxymethylated (OH) and hemimethylated (HM) substrates together in one reaction mixture, thus allowing to determine methylation rates in competitive settings. This gives the enzyme the full choice of substrates and ensures that technical parameters like enzyme or AdoMet concentrations are identical for all substrates. In the next step of our work, we systematically studied the methylation of long DNA substrates which were either unmethylated, hemimethylated or contained a pattern of un- and hemimethylated CpG sites thus mimicking biological substrates in cells after DNA replication. Similar experiments were conducted with a DNMT1 mutant with inactivated CXXC domain (19) to uncover the potential influence of this domain in the specificity of DNMT1.

Our data demonstrate that DNMT1 has a strong but flanking sequence dependent HM/UM specificity of 80-fold on average on single-site substrates which is slightly enhanced on long DNA substrates with methylation patterns. The HM/OH preference of DNMT1 is flanking sequence dependent as well but on average only 13-fold, indicating that passive loss of DNA methylation by 5hmC generation and inhibition of DNMT1 is not an efficient process in many flanking contexts. Our data show that the DNMT1 CXXC domain has a moderate flanking sequence dependent contribution to the HM/UM specificity of DNMT1 during the binding process of DNA to DNMT1, but not if DNMT1 slides along the DNA molecules and methylates several CpG sites in a processive methylation mode. Flanking sequence preferences on the single-site substrates were similar for HM and OH substrates, while differences were observed for UM substrates. Comparison of genomic methylation patterns from mouse ES cell lines with various deletions of DNMTs and TETs with our data revealed that the UM specificity profile is most related to cellular methylation patterns, indicating that *de novo* methylation activity of DNMT1 shapes the methylome in these cells.

## MATERIALS AND METHODS

### Expression and purification of DNMT1 for biochemical work

Full length murine DNMT1 (UniProtKB P13864, https://www.uniprot.org/uniprot/P13864) was overexpressed and purified as described (26) using the Bac-to-Bac baculovirus expression system (Invitrogen). The expression construct of DNMT1 with the mutated CXXC domain was taken from Bashtrykov *et al.*, 2012 (19).

### Synthesis of the long DNA substrates and methylation reactions with them

The sequence of the 349 bp substrate with 44 CpG sites was taken from Adam *et al.*, 2020 (26). Three different substrates were used that contained internal 3-nucleotide sequence tags to distinguish them after mixing in the sequencing analysis (Supplemental Figure S1). The sequence differences were at least 4 base pair away from the nearest CpG site. In case of the unmethylated substrate, two additional CpG sites were generated that were not included in the analysis. Generation of the substrates and the methylation reactions were conducted as described (Supplemental Figure S2) (26). In brief, for the generation of hemimethylated substrates, the unmethylated DNA was methylated *in vitro* by M.SssI (purified as described (26)) to introduce methylation at all CpG sites, or by M.HhaI (NEB) together with M.HpaII (NEB) to introduce methylation at GCGC and CCGG sites. For the synthesis of the two substrates containing hemimethylated CpG sites, the upper strand of the methylated substrate was digested with lambda exonuclease, the single-stranded-DNA was purified and finally double-stranded hemimethylated DNA was generated by primer extension using Phusion^®^ HF DNA Polymerase (Thermo). Methylation reactions were conducted using mixtures of UM, fully hemimethylated and patterned substrate (total DNA concentration 200 ng in 20 μl) in methylation buffer (100 mM HEPES, 1 mM EDTA, 0.5 mM DTT, 0.1 mg ml^−1^ BSA, pH 7.2 adjusted with KOH) containing 1 mM AdoMet. DNMT1 concentrations and incubation times are indicated in the text. Methylation was followed by bisulfite conversion using the EZ DNA Methylation-Lightning™ Kit (ZYMO RESEARCH) followed by library generation and Illumina paired-end sequencing (Novogene).

### Flanking sequence preference analysis with randomized single-site substrates

Methylation reactions of the randomized substrates with DNMT1 were performed similarly as described (26,29). Briefly, single-stranded oligonucleotides containing a methylated, hydroxymethylated or unmethylated CpG site embedded in a 10 nucleotide random context on either side were obtained from IDT and used for generation of 67 bps long double-stranded DNA substrates by primer extension (Supplemental Figure S3). An additional barcode was included outside of the randomized part to distinguish the three substrates after mixing in the sequencing analysis (Supplemental Figure S4). Pools of these randomized substrates were then mixed in different combinations, and methylated by DNMT1 in methylation buffer (100 mM HEPES, 1 mM EDTA, 0.5 mM DTT, 0.1 mg ml^−1^ BSA, pH 7.2 adjusted with KOH) containing 1 mM AdoMet. DNMT1 concentrations and incubation times are indicated in the text. Methylation was followed by hairpin-ligation, then bisulfite conversion was performed using the EZ DNA Methylation-Lightning™ Kit (ZYMO RESEARCH) followed by library generation and Illumina paired-end sequencing (Novogene).

### Bioinformatics analysis

NGS data sets were bioinformatically analyzed using a local instance of the Galaxy server (30) basically as described (26,31,32). In brief, for the long substrate, reads were trimmed, filtered by quality, mapped against the reference sequence and demultiplexed using substrate type and experiment-specific barcodes. Afterwards, methylation information was assigned and retrieved by home-made scripts. For the randomized substrate, reads were trimmed and filtered according to the expected DNA size. The original DNA sequence was then reconstituted based on the bisulfite converted upper and lower strands to investigate the average methylation state of both CpG sites and the NNCGNN flanks using home-made scripts. Methylation rates of 256 NNCGNN sequence contexts in the competitive methylation experiments with the mixed single-site substrates were determined by fitting to monoexponential reaction progress curves with variable time points with MatLab scripts as described (33). Pearson correlation factors were calculated with Excel using the correl function.

### Radioactive DNA methylation kinetics

Experimental validation of the determined flanking sequence preferences of DNMT1 was carried out using an avidin-biotin methylation plate assay as described (26) using oligonucleotides containing a single CpG site in UM, OH or HM state (Supplemental Table S1).

### Analysis of genomic DNA methylation patterns

DNA methylation in wildtype murine ES cells, as well as multiple DNMT and TET KO cell lines was investigated using whole genome bisulfite data published by Li *et al.*,2015 (GEO accession number GSE61457, data sets: GSM1505240-43) (34) which were processed as described (26). Data from Wang *et al.*, 2020 (GEO accession number GSE116482, data sets: GSM3239875, GSM3239876, GSM3239884, GSM4809269) (35) were filtered for coverage >4 only using the upper DNA strand. Average methylation levels in all NNCGNN flanks were determined with a home-written script. Correlations of genomic NNCGNN methylation patterns with DNMT1 preferences were based on Pearson *r*-value determined with the Excel correl function. For statistical analysis of the significance of correlations, genomic methylation profiles were randomized 20 times and the correlation analysis was repeated. In these randomizations, the measured genomic methylation levels were randomly assigned to NNCGNN sequences. Based on the average *r*-value of the randomized data sets and its standard deviation, *P*-values for the significance of the original correlations were determined by *Z*-statistics.

## RESULTS

### Flanking sequence preferences of DNMT1 for UM, OH and HM methylation

**Figure 1.**
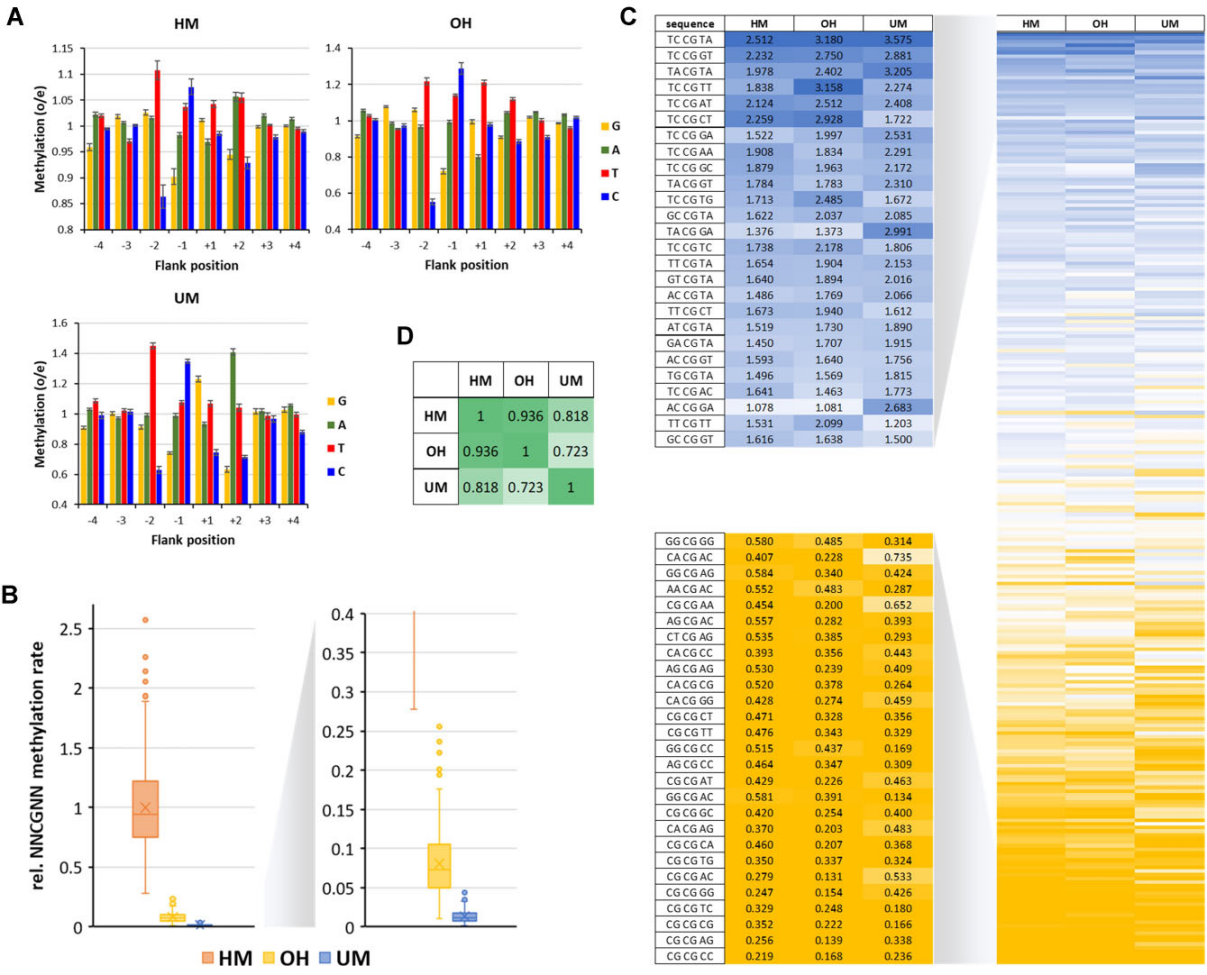
Analysis of flanking sequence effects on CpG methylation at HM, OH and UM sites. (**A**) Flanking sequence preferences of DNMT1 for the −4 to + 4 region. Profiles are based on the observed/expected ratio of nucleotide distribution in the methylated and unmethylated sequence reads. Shown are averages of profiles of individual time points, experiments and repeats and their SEM. *N* = 21 (including two data sets from Adam *et al.*, 2020) in case of HM, 18 in case of OH and UM. See also Supplemental Figure S6. (**B**) Box plots of the average reaction rates at all 256 NNCGNN sites. Boxes show the median, 1st and 3rd quartile. Whiskers display the 1.5 IQR distance. Outliers are indicated by dots. X indicates the average. (**C**) Heatmap of average methylation rates at the NNCGNN sites. (**D)** Correlation of average methylation rates at NNCGNN sites in different substrates. Shown are Pearson *r*-values.

In previous studies, strong flanking sequence preferences of DNMT1 on HM substrates were discovered (26), as well as flanking sequence preferences of DNMT3A (28,29), DNMT3B (29,32) and TET enzymes (33). However, the effect of flanking sequences on the specificity of DNMT1 has not yet been investigated. Therefore, we employed oligonucleotides containing one unmethylated (UM), hemihydroxymethylated (OH) or hemimethylated (HM) CpG site in a context of 10 randomized bases on either side as methylation substrates. Methylation reactions were conducted with HM/OH, HM/UM and OH/UM binary substrates mixtures as well as a HM/OH/UM ternary mixture. Using these 4 different settings, different DNMT1 concentrations and time points, in total 24 methylation data points comprising approximately 1766 million sequence reads were generated (Supplemental Table S2, Supplemental Figures S3 and S5). Control reactions without enzyme indicated very low levels of false methylation detection. In addition, reactions with hemimethylated randomized substrates conducted before (26) were included in the analysis. To obtain a first overview of the trends in the data sets, all methylated and unmethylated reads of each substrate type (HM, OH, or UM) were pooled and the distribution of A, T, G and C at the -8 to + 8 flanking base pairs in methylated and unmethylated sequences determined and compared (Figure 1A, Supplemental Figure S6). As observed in our previous study on HM substrates (26), the largest effects of the flanking sequences were detected between the –2 and + 2 sites. In general, similar observed/expected (o/e) patterns of bases were found with all three substrates (Table 1). On the 5′ side of the CpG, T(-2) was preferred and C(-2) disfavored, C(-1) was preferred and G(-1) disfavored. On the 3′ side, profiles differed in some details between HM/OH and UM. For OH and HM, T(+1) was preferred and A(+1) disfavored. In the case of UM, G(+1) was preferred and C(+1) disfavored. At the +2 site, HM/OH preferred A or T and disfavored G or C at this site, while UM showed a stronger preference for A(+2). The HM profiles are compatible with our previous findings and the similarity of all profiles indicates that the influences of the flanking base sequence on base flipping and DNMT1 conformations are to a large extent independent of the readout of the mC/hmC/C in the complementary strand.

**Table 1.** Summary of the flanking sequence preferences of DNMT1 for the methylation of HM, OH and UM substrates

	**–2**		**–1**		**+1**		**+2**	
	Favored	Disfavored	Favored	Disfavored	Favored	Disfavored	Favored	Disfavored
HM	T	C	C	G	T		A, T	
OH	T	C	C	G	T	A	A, T	
UM	T	G, C	C	G	G	C	A	C, G

### Analysis of the methylation rates of NNCGNN substrates

For a more detailed quantitative analysis and to determine real methylation rates, the average methylation levels of NNCGNN sites were determined for all different substrate types in all experiments. Next, for each mixture the different time points and enzyme concentrations were used for a fit of the methylation reaction progress of each individual NNCGNN site to a characteristic first order rate constant (k_NNCGNN_). Supplemental Figure S7A shows the pairwise correlation of the NNCGNN methylation profiles derived in the individual experiments (each of them based on methylation kinetics over several time points). Comparison of HM profiles derived from HM only, HM/UM, HM/OH, or HM/OH/UM reveals for example correlation factors between 0.92 and 0.89, OH profiles from different experiments show correlations of 0.96 and 0.97, UM profiles 0.87 and 0.92. These high pairwise correlations of profiles obtained for the same substrate type (HM, OH or UM) indicate that the independent rate fittings were reproducible and the results are reliable. Moreover, individual HM and OH profiles were also highly correlated, while the correlation of HM and OH profiles to UM profiles was lower (Supplemental Figure S7A). Next, the different methylation kinetics were scaled using the average methylation rates of the HM, OH and UM substrates in the individual reactions (Supplemental Figure S7B) and using these scaling factors, the rate constants determined in the different experiments were averaged. After normalization of the pairwise data, the individual rates of all 256 NNCGNN sites determined in the different pairwise settings were also compared. As shown in Supplemental Figure S7C, the SEM values of these average rates are small, around ±5% for the HM substrate and ±10% for OH and UM, indicating that they are well-defined. The slight increase in relative SEM is due to the fact that methylation rates drop when going from HM to OH and UM. The combined methylation rates of all 256 NNCGNN sequences in HM, OH and UM context, as well as their corresponding SEM values are provided in Data Set 1. The methylation rates of the HM substrate varied over a range of about 10-fold, variances of the OH and UM methylation rates were larger about 25-fold (Figure 1B). Exemplary methylation rates of favored and disfavored substrates were validated by radioactive kinetics using oligonucleotide substrates with defined sequences (Supplemental Figure S8).

**Figure 2.**
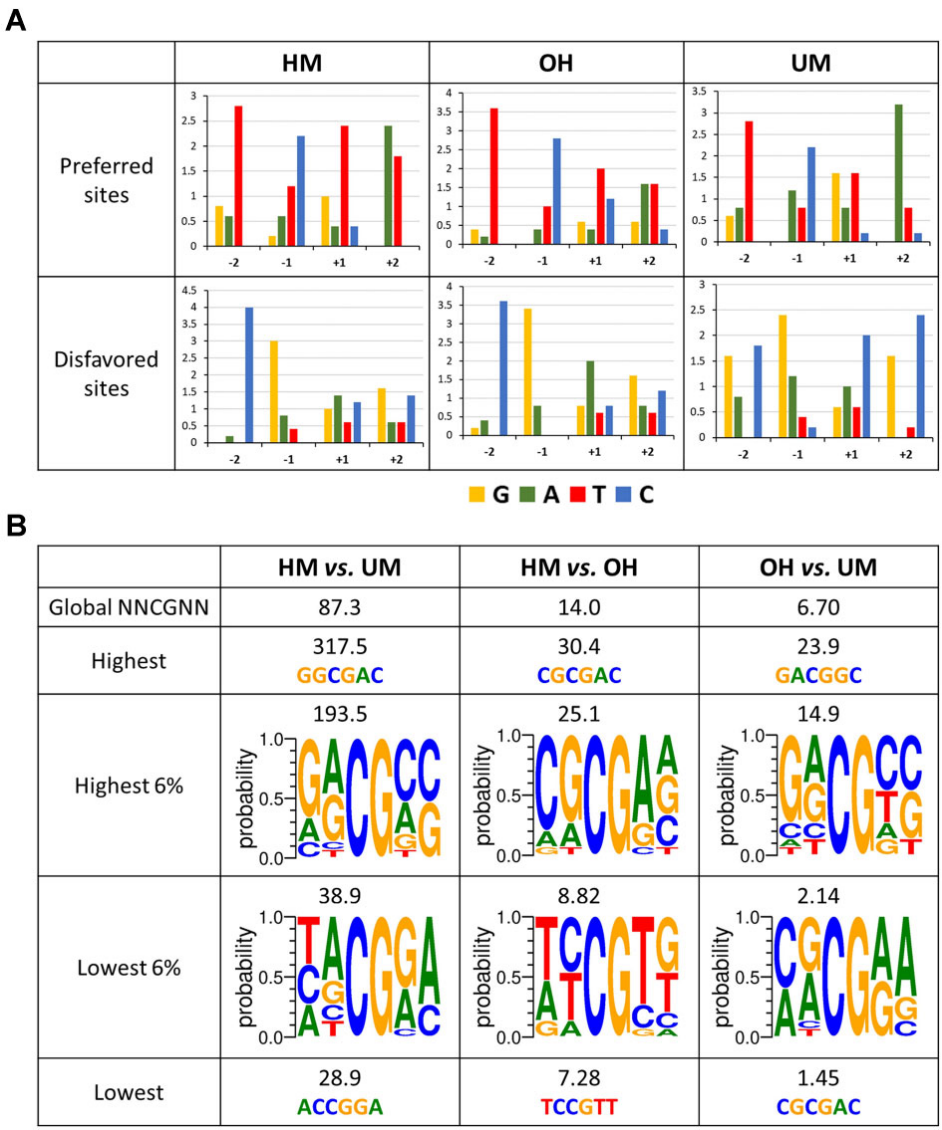
Sequence preferences and DNMT1 specificity. (**A**) Occurrence of bases in the –2 to +2 positions of the 20 most preferred and most disfavored NNCGNN flanks. Flanks were sorted based on the methylation rates of all 256 HM, OH and UM substrates determined in the exponential fitting of the methylation data. (**B**) Degree and sequence context of HM/UM, HM/OH and OH/UM ratio preferences of WT DNMT1 based on the NNCGNN methylation rates.

Comparison of the averaged HM, OH and UM methylation rates of corresponding NNCGNN substrates (Figure 1C) showed high correlation of HM and OH profiles, while correlation to UM was weaker (Figure 1D) as expected from the primary data correlations (Supplemental Figure S7A). We extracted from these quantitative data preferred and disfavored sequences in all sequence contexts (Figure 2A), revealing no big differences in the preferences in the HM and OH context, except a slightly enhanced disfavor for A(+1) on the OH substrate. In contrast, the UM profile showed more pronounced differences from HM, viz. a disfavor for G(-2), preference for G(+1), disfavor for C(+1), and disfavor for C(+2) and G(+2). These new specificity profiles are in good agreement with the published observation that UM activity of DNMT1 is high on CCGG substrates (16). Next, we were interested to compare the pairwise preferences of DNMT1 for individual substrates and calculated the pairwise ratio of the 256 k_NNCGNN_ for the HM, OH and UM substrates. As shown in Figure 2B, on average HM substrates are methylated 87-times faster than UM substrates. The HM/UM specificities range from >300 fold for GGCGAC, the substrate with the highest HM/UM ratio, to 29-fold for ACCGGA, the substrate with the lowest HM/UM specificity. However, even at this target site DNMT1 showed a decent almost 30-fold preference for HM. This is also illustrated by ranges of k_NNCGNN_ rates shown in Figure 1B, where the methylation rates of the most disfavored HM substrates are well separated from the rates observed at the best UM substrates.

Comparison of the OH and HM methylation rates revealed smaller differences, because OH substrates, on average, are methylated only 14-fold weaker than HM (range 30-fold to 7-fold) (Figure 2B). In this case it is noticeable that the methylation rates of most disfavored HM substrates are similar to the methylation rates of the most preferred OH substrates (Figure 1B). Hence, the global conclusion that DNMT1 is inactive on OH substrates cannot be made. This is also illustrated by the fact that OH substrates were methylated about 7-fold (range 24-fold to 1.5-fold) better than UM substrates.

### HM/UM specificity of DNMT1 in the methylation of long DNA molecules

**Figure 3.**
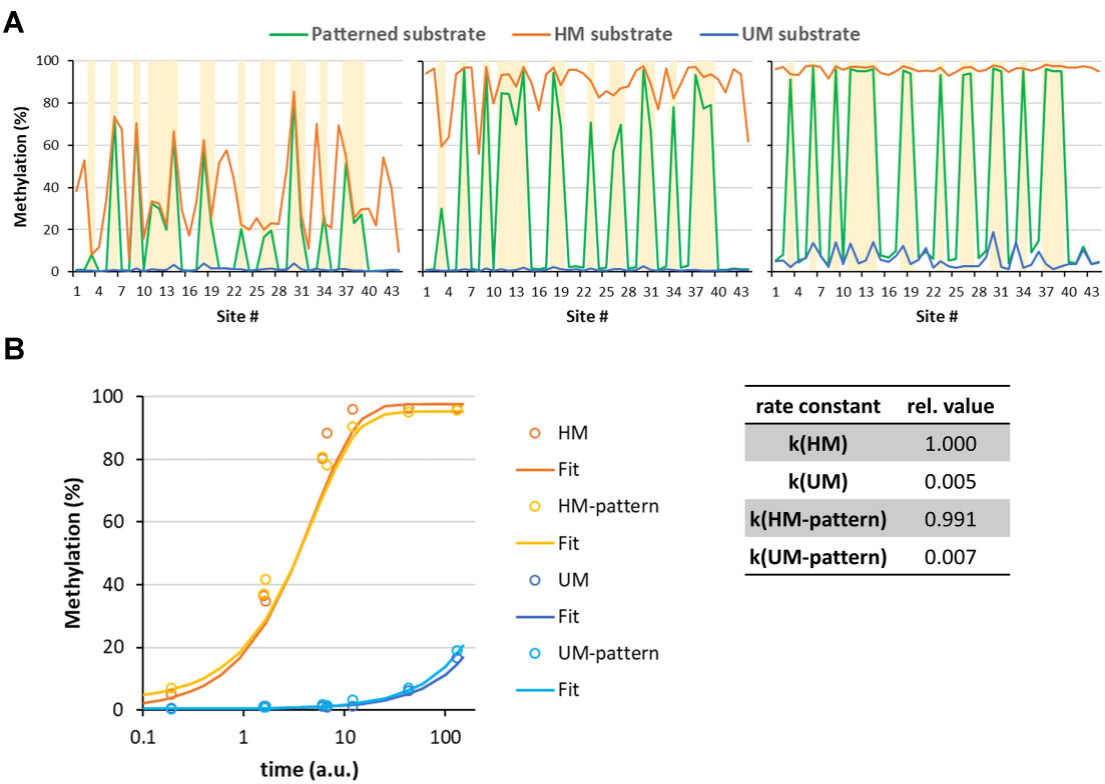
HM/UM specificity of the DNMT1 methylation reactions with mixed long HM, UM and patterned substrates. (**A**) Examples of methylation levels (see also Supplemental Figure S9). Yellow shading highlights the hemimethylated sites on the patterned substrate. (**B**) Average methylation rates of HM and UM substrates and HM as well as UM sites on the patterned substrate. UM, completely unmethylated substrate; HM, substrate only containing hemimethylated CpG sites; patterned substrate is hemimethylated at GCGC and CCGG sites and unmethylated at all other CpG sites.

Next, we were interested to investigate the specificity of DNMT1 on a long DNA substrate with 44 CpG sites, which mimics natural DNA substrates appearing in cells after DNA replication (Supplemental Figure S2). The substrate was used in an unmethylated form, completely hemimethylated at all CpG sites and with a pattern of 18 hemimethylated CpG sites (all sites in CCGG and GCGC context) and 26 unmethylated sites (all other sequence contexts). We then methylated a mixture of all three long DNA substrates with DNMT1 and followed the kinetics of methylation at two enzyme concentrations over 4 time points by bisulfite conversion and NGS (Figure 3A, Supplemental Table S3, Supplemental Figure S9). The obtained data clearly showed a very efficient global methylation of the hemimethylated DNA, weak methylation of the unmethylated DNA and specific methylation of the patterned DNA at the hemimethylated CpG sites (Figure 3B). We extracted the methylation levels of the HM, UM and patterned substrate, the latter separated for hemimethylated CpG sites and unmethylated CpG sites and fitted the individual data points to a reaction progress curve to determine the respective average methylation rates. As shown in Figure 3B, the methylation of hemimethylated sites on the HM and patterned long DNA substrate were almost identical. Similarly, the methylation rates of unmethylated CpG sites on the UM and patterned DNA were comparable. The overall ratio of the methylation of sites on the HM and UM substrates was about 180, and the HM/UM on the patterned substrate (145) was similar. Based on the flanking sequences of the methylation sites, a preference of 126 would have been expected for HM versus UM and 102 for the methylation of the sites on the patterned substrate. This result shows that the specificity of DNMT1 is slightly enhanced on the longer DNA substrate with more target sites than on single-site substrates.

Next, we wanted to compare the relative specificities observed on the fully HM vs. fully UM substrates with the methylation of HM and UM sites on the patterned substrate. When considering the expected specificity ratio, the relative specificities observed in these two reactions are identical (observed: HM/UM ratio = 180, patterned HM/UM ratio = 145, ratio 180/145 = 1.241, expected HM/UM specificities for both substrates 126/102 = 1.235). This finding indicates that the relative HM/UM specificity of DNMT1 is influenced by neither the presence of additional CpG sites on the same DNA molecule nor their methylation state.

### Role of the DNMT1 CXXC domain for HM/UM specificity

**Figure 4.**
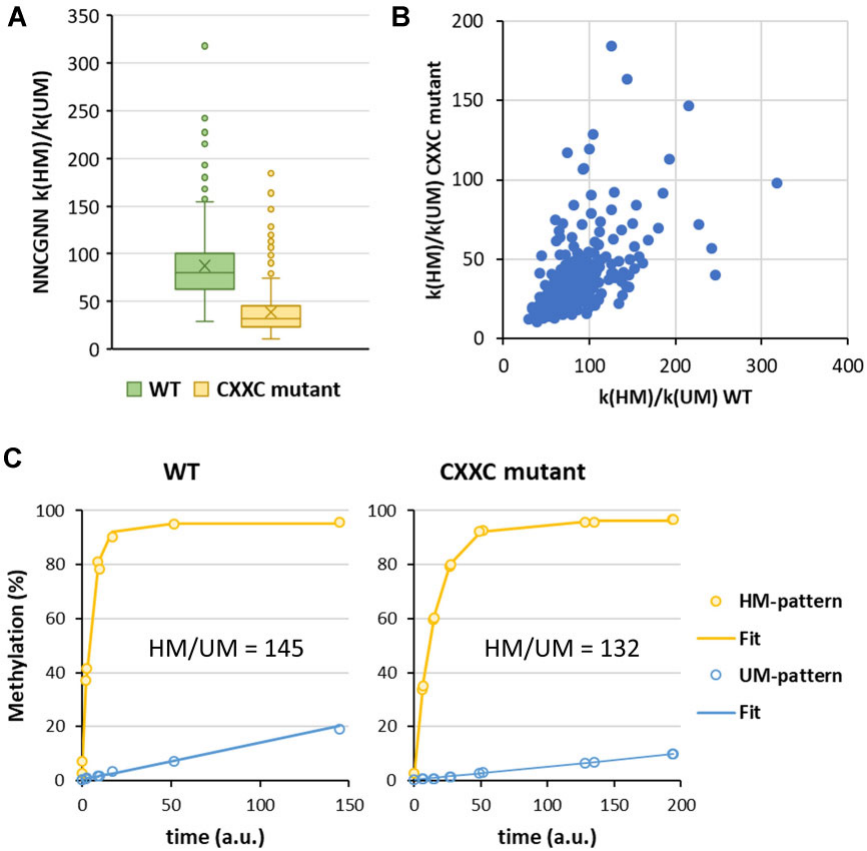
HM/UM specificity of the CXXC mutant DNMT1. (**A**) Compilation of NNCGNN methylation rates determined on HM and UM substrates with single CpG sites in randomized flanking context. Boxes show the median, 1st and 3rd quartile. Whiskers display the 1.5 IQR distance. Outliers are indicated by dots. X indicates the average. (**B**) Comparison of the methylation rates of WT DNMT1 and CXXC mutant on each NNCGNN substrate. See also Supplemental Figure S11. (**C**) Fits of the methylation of HM and UM sites on the long patterned DNA substrate by WT DNMT1 or the CXXC mutant.

Next, we aimed to dissect the role of the CXXC domain in the HM/UM specificity of DNMT1. For this, we used a DNMT1 mutant containing 4 amino acid exchanges in the CXXC domain that completely abrogate DNA binding of this domain (19). To test the effect of the CXXC domain in all possible flanking sequence contexts, unmethylated and hemimethylated substrates containing a single CpG site in randomized sequence context were mixed and methylated by the CXXC mutant (Supplemental Figure S10, Supplemental Table S4). The data were analyzed as described above regarding the methylation levels of all NNCGNN sites and compared with the HM and UM data of the WT DNMT1 experiments. The direct comparison of the mutant and WT methylation rates at all 256 sites revealed a highly significant (*P*-value 8.66 × 10^−67^, based on two-sided t-test of paired data) but mild about 2.5-fold reduced HM/UM specificity of the CXXC mutant (Figure 4A). However, the effect was highly sequence dependent with most sites showing an about 2-fold effect, but at some sites no effect was observed while others showed up to 6-fold changes (Figure 4B).

It is noticeable that the CpG substrate used in the paper which reported a strong effect of the CXXC domain in the HM/UM specificity of DNMT1 (27) contained a CpG site with flanks among the most strongly affected ones. In contrast, the flanks of the CpG site in the substrate used in the paper which did not report strong effects of the CXXC domain (19) were among the moderately affected ones (Supplemental Figure S11). Hence, the different substrates used in both studies can partially explain their diverging findings. Another difference between both studies was that a truncated DNMT1 was used in one of them and full-length DNMT1 in the other, which may affect the domain movements.

Furthermore, we investigated the specificity of the CXXC mutant on the patterned long substrate. A methylation kinetic was determined (Supplemental Table S4, Supplemental Figure S12A) and the methylation rates of HM and UM sites were extracted and compared with the corresponding data obtained with WT DNMT1 (Supplemental Figure S12B, extracted from Supplemental Figure S9). The overall methylation rates of HM and UM sites on the patterned substrate revealed a 132-fold ratio of HM/UM for the CXXC mutant (Figure 4C), which is almost identical to the value observed with WT DNMT1. Hence, while the CXXC mutant DNMT1 showed a significant about 2.5-fold reduced HM/UM specificity on single-site substrates (Figure 4A), no noticeable effect was detected on the long substrate with methylation pattern (ratio HM/UM 145 for WT and 132 for CXXC).

### Comparison of DNMT1 specificity profiles with cellular DNA methylation levels

**Figure 5.**
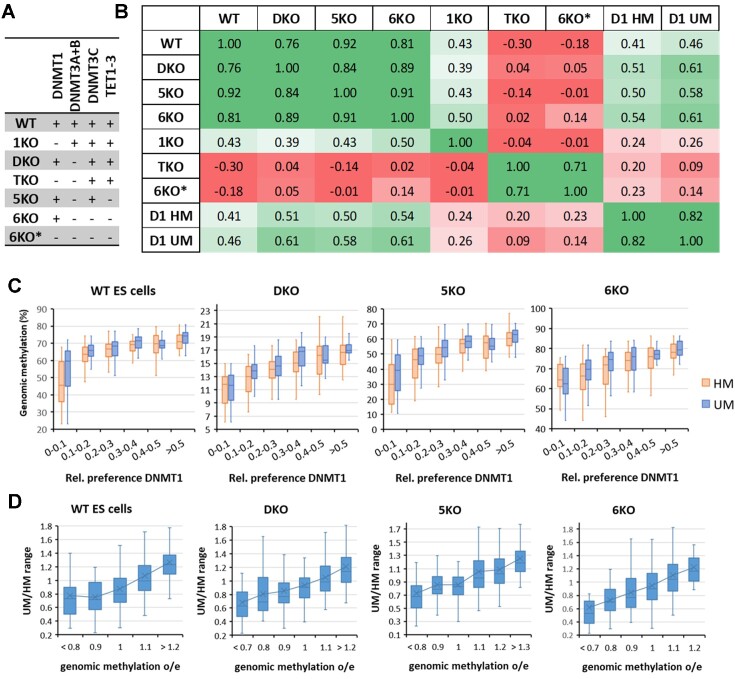
DNMT1 UM flanking sequence preferences shape cellular methylation patterns. (**A**) Compilation of cell lines used in this analysis. WT, 1KO, DKO and TKO data were taken from Li *et al.*, 2015 (34), 5KO, 6KO and 6KO* date were taken from Wang *et al.*, 2020 (35). (**B**) Correlation of the cellular NNCGNN methylation levels with the DNMT1 HM (D1 HM) and UM (D1 UM) preferences. Shown are Pearson *r*-values. (**C**) Correlation of cellular NNCGNN methylation levels of cell lines containing DNMT1 with DNMT1 flanking sequence preferences. (**D**) Correlation of UM/HM ratios of DNMT1 methylation preferences with genomic methylation levels of cell lines containing DNMT1. Boxes in panels C and D show the median, 1st and 3rd quartile. Whiskers display the 1.5 IQR distance. X indicates the average.

Finally, we aimed to investigate, if the newly determined DNMT1 methylation preferences affect cellular DNA methylation patterns. For this, we re-analyzed published genome-wide DNA methylation data from various mouse ES cells lines with deletions of DNMTs and TETs (Figure 5A) (34,35). In our previous work, we showed that the NNCGNN methylation profiles of the WT and DKO cells are strongly correlated with DNMT1 HM flanking sequence preferences (26). We now extended this analysis to the UM profile and included the following mouse ES cell lines: WT, 1KO (DNMT1 KO), DKO (DNMT3A and DNMT3B KO), TKO (DNMT1, DNMT3A and DNMT3B KO), 5KO (DNMT3A, DNMT3B and TET1-3 KO), 6KO (DNMT3A, DNMT3B, DNMT3C, and TET1-3 KO), and 6KO* (6KO with additional DNMT1 KO) (Figure 5A). Overall, NNCGNN correlations of genomic methylation levels and the DNMT1 preferences (Figure 5B) revealed 4 well-defined clusters: (i) cell lines containing DNMT1 (WT, DKO, 5KO and 6KO), (ii) cell lines lacking DNMT1, but still containing DNMT3 enzymes (1KO), (iii) cell lines lacking DNMT1, DNMT3A and DNMT3B (TKO, 6KO*) and (iv) the UM and HM activity profiles of DNMT1.

Strikingly, in all data sets, the activity profiles of DNMT1 were strongly correlated with methylation patterns of cell lines containing DNMT1 (Figure 5B and C). Statistical analysis based on 20 data sets with randomized genomic methylation profiles revealed *P*-values of the correlation of the genomic methylation profiles with the DNMT1 HM and UM profiles to occur by chance of <5 × 10^−12^ in each case. Interestingly, in all these cases the correlation was stronger with the UM profile (Figure 5B). Statistical analysis using the same approach revealed *P*-values for the better correlation of cellular methylation patterns with the DNMT1 UM profile (when compared with HM) of 0.021, 4.4 × 10^−3^, 8.5 × 10^−3^ and 7.7 × 10^−3^ for WT, DKO, 5KO and 6KO cells, respectively. The high significance of this effect is also illustrated by the fact that an improved correlation of cellular methylation profiles to the UM profile of DNMT1 was observed in 4 independent cell lines with different genetic background based on data provided by different labs. In contrast, no or weak correlations were observed between methylation patterns of cells not containing DNMT1 and the DNMT1 preferences (Figure 5A). The impact of the UM preference on cellular methylation patterns is also illustrated by plots of the DNMT1 UM/HM ratios of the NNCGNN methylation rates against the o/e levels of genomic methylation in the 256 NNCGNN sequence contexts, showing that genomic methylation levels are strongly correlated with UM/HM ratios (Figure 5D). Of note, these effects are not only seen in ‘DNMT1-only’ cell lines (DKO, 5KO, 6KO) but also, albeit to a lower extent, with WT ES cells.

## DISCUSSION

The specificity of DNMT1 for hemimethylated DNA is a central feature of the inheritance of DNA methylation and its function as a heritable epigenetic signal. We investigated the flanking sequence dependence of this specificity in a comprehensive manner using Deep Enzymology experiments with HM, OH and UM substrates investigating CpG sites in all possible NNCGNN flanks. Methylation kinetics of different substrates were conducted in a competitive setting and they provided sufficient kinetic resolution. Experiments were conducted in independent repeats using different incubation times, enzyme concentrations and also mixtures to extract a set of 768 rate constants describing the activity of DNMT1 on all 256 NNCGNN sites in an UM, OH and HM context. Flanking sequence preferences of DNMT1 on HM and UM substrates are related, but distinct from preferences of other DNMT and TET enzymes (Supplemental Figure S13 based on additional data taken from (29,31–33)). Our data show that the HM/UM specificity of DNMT1 is around 100-fold, but it varies about 10-fold with the flanking sequence. Our data are in general agreement with previous findings (12–20), but they provide a global picture including the differences in HM/UM specificity between different CpG sites.

This specificity is comparable on single CpG site substrates and long DNA molecules containing patterns of methylated and unmethylated CpG sites. The relative HM/UM specificity of DNMT1 was not influenced by the presence of additional CpG sites on the same DNA molecule or their methylation state. However, it was slightly enhanced on the longer DNA substrate with more target sites than on single-site substrates. This observation can be explained, because in the methylation of single-site substrates product dissociation in multiple turnovers affects the overall rates, but this step does not contribute to the HM specificity. In contrast, on long multi-site substrates DNMT1 can slide along the DNA without dissociation after one methylation event, hence the overall specificity can be higher.

A 100-fold preference for HM DNA corresponds to a lowering of the transition state energy of the methylation reaction (Δ*G*^#^) by about 11 kJ/mol. This is a remarkable property given that the preferred target site is defined by the mere presence of a single methyl group. How can this strong discrimination be explained? The solvent accessible surface of a pyrimidine C5-methyl group in DNA is 30.3 Å² (36) and the energy associated with the burial of hydrophobic surface area is around 60.8 J/mol and Å² (37). Hence, the interaction of the C5-methyl group with the hydrophobic pocket in the active sites of DNMT1 is expected to contribute about 1.9 kJ/mol to ΔΔ*G*^#^, much less to explain the 100-fold difference in reaction rates. The only mechanism strong enough to translate the presence of a single methyl group into rate enhancements of 100-fold is steric repulsion. Hence, we propose a model in which DNMT1 binds to UM substrates in an inactive conformation. The presence of the 5mC methyl group creates a steric overlap and pushes the conformation of the complex into an active state from which base flipping and catalysis occurs. Future studies will show, if this hypothetical inactive binding mode of UM substrates can be structurally identified and characterized.

An HM/UM specificity of 100-fold means that on average every hundredth unmethylated CpG site the enzyme encounters will aberrantly be methylated. We show that this preference is sufficient to copy an existing methylation pattern on a long DNA molecule with fairly high accuracy *in vitro*. *In vivo*, DNMT1 may transfer only very few methyl groups into a typical unmethylated CGI, which afterwards can be easily removed by TET enzymes found at CGIs (22) to keep them unmethylated. However, the 100-fold HM/UM preference is not sufficient to copy the exact methylation state of all 56 million CpG sites in the diploid human genome. Hence, the maintenance of the DNA methylation pattern also relies on crosstalk with other chromatin modifications as shown previously (2,38–40).

The role of the DNMT1 CXXC domain in the specificity of DNMT1 was unclear, due to conflicting experimental data (19,27). We show here that the CXXC domain has a moderate flanking sequence dependent contribution to HM/UM specificity on single CpG site substrates which is in the range of about 2.5-fold on average. Flanking sequence effects could partially resolve diverging literature data. However, the effect of the CXXC domain on the HM/UM specificity was only detectable with the single CpG substrates, but not on long DNA substrate with multiple CpG sites. This finding suggests a model in which DNA entering the DNA binding tunnel of DNMT1 encounters the CXXC domain, allowing CXXC to prevent the binding of DNA containing UM CpG sites. After the initial binding and the conformational changes towards a closed conformation (26), DNMT1 can move along the DNA and methylate several sites in a processive manner (16,17,26). During this reaction, the DNA presumably stays in the central binding tunnel and it does not come in contact with the CXXC domain again, explaining the lack of influence of the CXXC domain on the specificity of DNMT1 on long multi-site DNA substrates.

Inhibition of DNMT1 on DNA substrates containing hemihydroxymethylated CpG sites has been discussed as a potential mechanism for passive DNA demethylation. To uncover the fundamental parameters determining this process, we also determined the HM/OH preference of DNMT1. We observed that it is flanking sequence dependent and on average only 13-fold. In fact, the most preferred OH sites are methylated at similar rates as the least preferred HM sites. This suggests that the efficiency of passive DNA demethylation of hydroxymethylated DNA by inhibition of DNMT1 depends on the sequence context, and in many sequence contexts it is expected not to be very efficient. This finding suggests that mCpG/hmCpG dyads should exist in genomic DNA. Regarding the pathways of DNA demethylation, based on our data, TET mediated oxidation of 5-methylcytosine to higher oxidation forms followed by their removal catalyzed by TDG (11) is likely to play a more important role than passive loss of DNA methylation by inhibition of DNMT1 on hemihydroxymethylated CpG dyads in many sequence contexts.

Finally, we observed that flanking sequence preferences of DNMT1 on unmethylated substrates partially differ from preferences on HM and OH substrates. Comparison of genomic methylation patterns from mouse ES cell lines with various deletions of DNMTs and TETs with our data revealed that the UM specificity profile of DNMT1 is most related to cellular methylation patterns of cells only containing DNMT1 as active DNMT. We observed in three unrelated cell lines in which DNMT3A and DNMT3B are deleted but DNMT1 is still present that their residual DNA methylation is more correlated with the DNMT1 UM than HM profile, indicating that *de novo* activity of DNMT1 is needed to compensate the loss of methylation caused by the KO of the DNMT3 enzymes. Similar effects, albeit to a weaker extent, were also observed in WT ES cells. These observations suggest that the *de novo* methylation activity of DNMT1 on unmethylated DNA is the limiting reaction for DNA methylation in DNMT1-only cells, but it also affects cellular methylation patterns in WT cells, in agreement with recent papers providing evidence for this type of activity in mouse ES cells (35,41). Based on our data, this effect represents a fundamental principle affecting cellular methylation patterns, but the detailed consequences of this require further studies.

Our findings illustrate how detailed flanking sequence preference analysis can reveal footprints of DNMT activity providing novel information about the function of DNMTs in living cells. In general, the rate of DNA methylation loss at particular CpG sites in a defined genomic locus during cell division is expected to depend on the sequence preferences of the DNMTs and TETs and their local activities at the genomic locus which are determined by their global expression levels, regulation, and the local targeting efficiency of each enzyme. Further studies will be needed to determine the local activities of all DNMTs and TETs at given genomic loci which finally should allow to model DNA methylation dynamics with CpG site resolution.

## Supplementary Material

gkad465_Supplemental_Files

## Data Availability

Global DNA methylation levels in mouse ES cell lines were taken from published whole genome bisulfite data (GEO accession number GSE61457, data sets: GSM1505240-43 (34), and GEO accession number GSE116482, data sets: GSM3239875, GSM3239876, GSM3239884, GSM4809269 (35)). The code used for genomic DNA methylation analysis is available at https://github.com/PavelBashtrykov/DNMT1_accuracy (permanent DOI: https://doi.org/10.6084/m9.figshare.22793609.v1). Sequences extracted from the NGS kinetic raw data generated in this study are available at DaRUS under https://doi.org/10.18419/darus-3334. Data Set 1 compiling the methylation rates of all 256 NNCGNN sequences in HM, OH and UM context, as well as their corresponding SEM values is available as an attachment to this paper and at DaRUS under https://doi.org/10.18419/darus-3334. The biochemical data underlying this article are available in the article and in its online supplementary material. All other data are available from the corresponding author upon reasonable request.
