# Supplementary material for: On the accuracy of the epigenetic copy machine: comprehensive specificity analysis of the DNMT1 DNA methyltransferase
Source: Nucleic Acids Res. 2023 May 29;51(13):6622–33. doi: 10.1093/nar/gkad465 (PMC10359454; doi:10.1093/nar/gkad465)
Supplement: gkad465_Supplemental_Files [file gkad465_supplemental_files.zip › D1_suppl_rev3.pdf]

On the accuracy of the epigenetic copy machine - comprehensive specificity analysis of the DNMT1 DNA methyltransferase

Sabrina Adam, Viviane Klingel, Nicole E. Radde, Pavel Bashtrykov & Albert Jeltsch*

Supplemental Information

Supplemental Figures

Supplemental Figure 1: Sequences of the long DNA substrates.
Supplemental Figure 2: Scheme of the Deep enzymology methylation experiments with long substrates here illustrated for the HM substrate containing only hemimethylated CpG sites.
Supplemental Figure 3: Scheme of the Deep Enzymology experiments conducted here.
Supplemental Figure 4: Sequences of the randomized single CpG site substrates.
Supplemental Figure 5: Compilation of the WT DNMT1 methylation reactions on libraries of HM, OH, and UM substrates with randomized -10 to +10 flank.
Supplemental Figure 6: Flanking sequence preferences of DNMT1 for the −8 to +8 region on HM, OH and UM substrates. Profiles are based on the observed/expected ratios of nucleotide distribution in the methylated and unmethylated sequence reads.
Supplemental Figure 7: Data processing of individual experiments.
Supplemental Figure 8: Experimental validation of substrate preferences by radioactive DNA methylation kinetics using two model substrates in HM, OH and UM state.
Supplemental Figure 9: Profiles of the DNMT1 methylation reactions with mixed long HM, UM and patterned substrates sorted by reaction progress.
Supplemental Figure 10: Methylation reactions on libraries of mixed HM and UM substrates with randomized -10 to +10 flank carried out with the DNMT1 CXXC mutant.
Supplemental Figure 11: Comparison of the methylation rates of WT DNMT1 and CXXC mutant on each NNCGNN substrate (taken from Figure 4B).
Supplemental Figure 12: Profiles of the CXXC mutant methylation reactions with long patterned substrates.

Supplemental Tables

Supplemental Table 1: Oligonucleotides used in the radioactive methylation kinetics.
Supplemental Table 2: Sequencing statistics for the methylation experiments of single CpG site substrates in randomized sequence context.
Supplemental Table 3: Sequencing statistics for the methylation experiments with long DNA substrates.
Supplemental Table 4: Sequencing statistics for the methylation experiments with the DNMT1 containing mutations in the CXXC domain.

Supplemental References

Data Set 1 compiling the methylation rates of all 256 NNCGNN sequences in HM, OH and UM context in tabular format, as well as their corresponding standard error of the mean (SEM) values, is available at as a separate attachment and at DaRUS under https://doi.org/10.18419/darus-3334.

Supplemental Figures

Supplemental Figure 1: Sequences of the long DNA substrates.

>long patterned
CCTGCCATGAAATCCCTCAAGTCTCCCCTCAGGGAACGCTGAAGCCGCGCCACGCCCCCGTCCTTACCAGTCCGGATC
AGCTGCTGTTCGCGAGCTGCCGGCCACGCACCAGCCCCGGAGGCGCTCCCGGGGCACAGCCGGCGGCGACTACGCCT
CCTCAGGCCCCCGGCGCCCGCCGACGCGCACGCCTCCACACGCGCGCGTCCAGTGGAGACCTGCGATTGGCTGCCAGG
TGCCGGCGCGAGATCGGCGCGGCTCCGAGCTAGGAGCATGCGCGCGCTCTGACGCCCCTGGTGGCGACGGCTGGAC
GCGGGGTTAAATTGAGAAGGAGGAGGGCAGCAGCAATACCC

>long HM var1
CCTGCCATGAAATCCCTCAAGTCTCCCCTCAGGGAACGCTGAAGCCGCGCCACGCCCCCGTCCAATCCAGTCCGGATC
AGCTGCTGTTCGCGAGCTGCCGGCCACGCACCAGCCCCGGAGGCGCTCCCGGGGCACAGCCGGCGGCGACTACGCCT
CCTCAGGCCCCCGGCGCCCGCCGACGCGCACGCCTCCACACGCGCGCGTCCAGTGGAGACCTGCGATTGGCTGCCAGG
TGCCGGCGCGAGATCGGCGCGGCTCCGAGCTAGGAGCATGCGCGCGCTCTGACGCCCTGAGTGGCGACGGCTGGAC
GCGGGGTTAAATTGAGAAGGAGGAGGGCAGCAGCAATACCC

>long UM var2
CCTGCCATGAAATCCCTCAAGTCTCCCCTCAGGGAACGCTGAAGCCGCGCCACGCCCCCGTCCGTGCCAGTCCGGATC
AGCTGCTGTTCGCGAGCTGCCGGCCACGCACCAGCCCCGGAGGCGCTCCCGGGGCACAGCCGGCGGCGACTACGCCT
CCTCAGGCCCCCGGCGCCCGCCGACGCGCACGCCTCCACACGCGCGCGTCCAGTGGAGACCTGCGATTGGCTGCCAGG
TGCCGGCGCGAGATCGGCGCGGCTCCGAGCTAGGAGCATGCGCGCGCTCTGACGCCCGATGTGGCGACGGCTGGAC
GCGGGGTTAAATTGAGAAGGAGGAGGGCAGCAGCAATACCC

The 44 CpG sites are highlighted yellow. In the unmethylated substrate, two additional CpG sites were generated by the sequence bar code that were not included in the analysis
The sequence tags used to distinguish the sequences are colored red.

Supplemental Figure 2: Scheme of the Deep enzymology methylation experiments with long substrates here illustrated for the HM substrate containing only hemimethylated CpG sites.

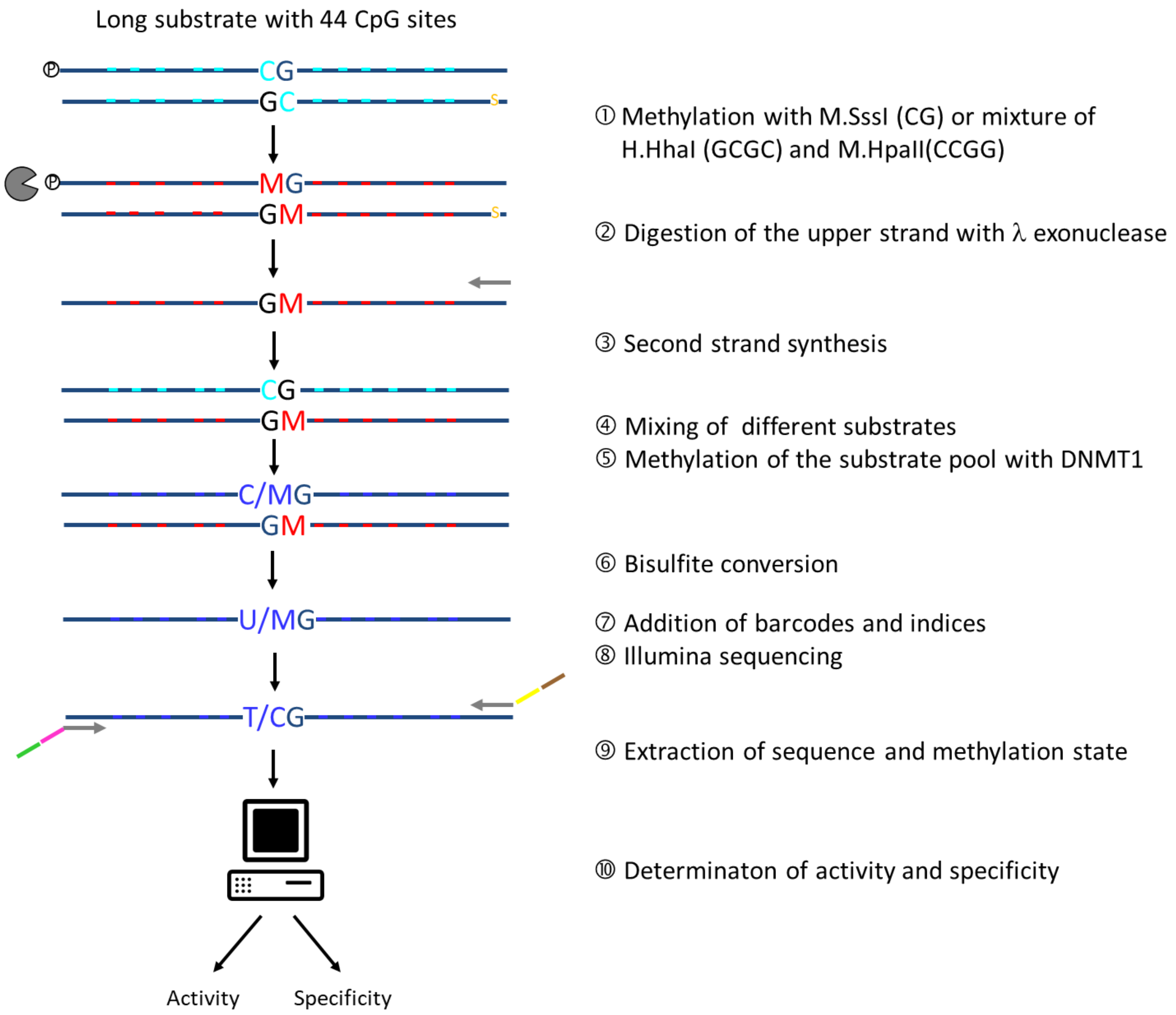

Supplemental Figure 3: Scheme of the Deep Enzymology experiments with random flank single-site substrates conducted here.

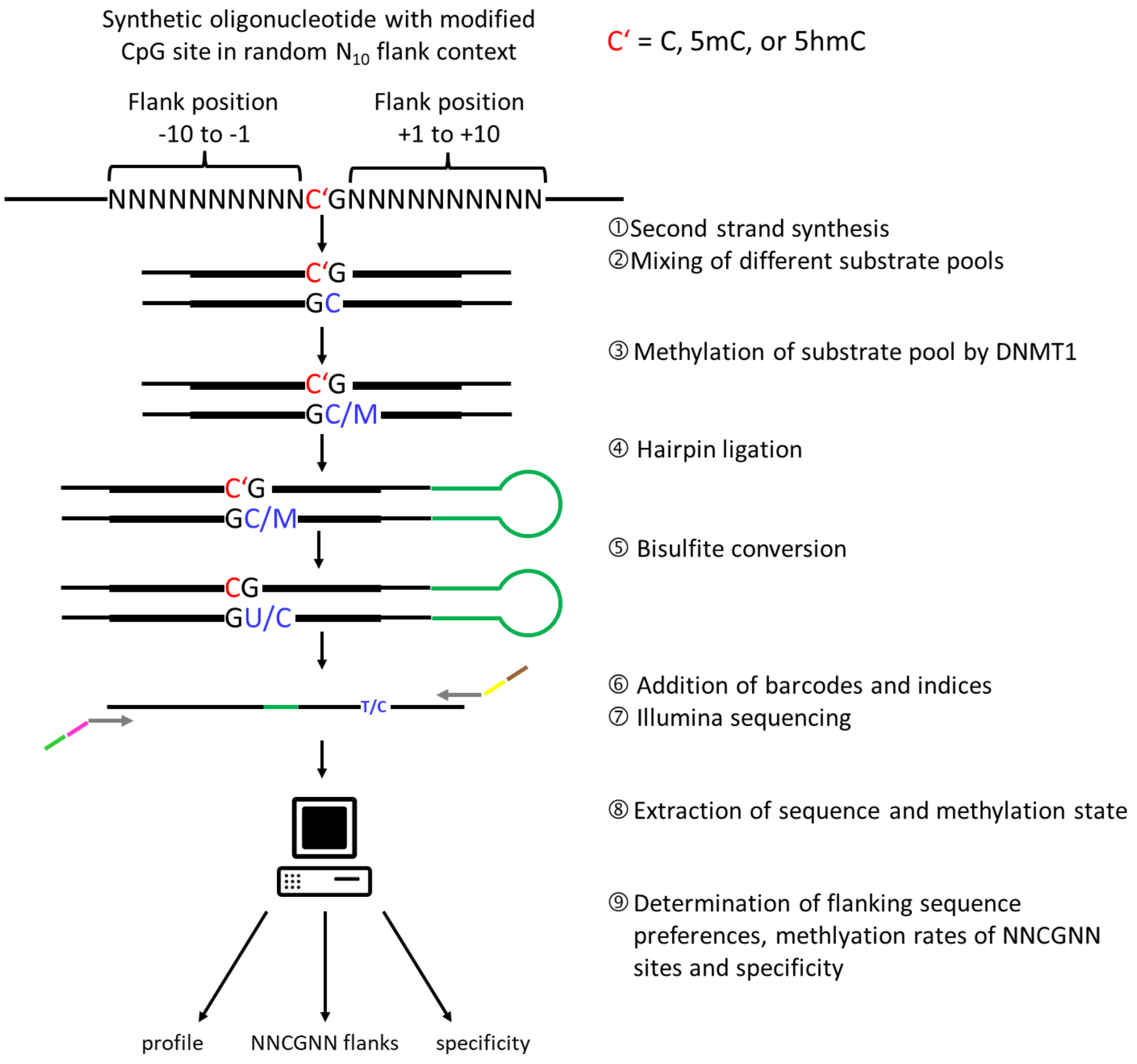

Supplemental Figure 4: Sequences of the random flank single-site substrates.

UM-substrate

GAGTGTGATTAGGTTTTTATTGTTTATGATTTTTCGATTTGTTTGTTGAGAAGGGATGTGGATATATATTTTTTTTTTGTAAATAGATCGGAAAATTATAGGT
AGTGAGAGTTTAGTTATATTT

OH-substrate

GAGTGTGATTAGGTTTTTATTGTTTATGATTTTT5hmCGATTTGTTTGTGAGAAGGGATGTGGATATATATTTTTTTTTTGTAAATAGATCGGAAAATTATAGG
TAGTGAGAGTTTAGTTATATTT

HM-substrate

GAGTGTGATTAGGTTTTTATTGTTTATGATTTTT5mCGATTTGTTTGTTAGAGAAGGGATGTGGATATATATTTTTTTTTTGTAAATAGATCGGAAAATTATA
GGTAGTGAGAGTTTAGTTATATTT

Random nucleotides

Random nucleotides

Hairpin

Sequence tag for discrimination of substrates in mixture

CpG site (modified strand)

CpG site (strand used for analysis)

Supplemental Figure 5: Compilation of the WT DNMT1 methylation reactions on libraries of HM, OH, and UM substrates with randomized -10 to +10 flank. Shown are average methylation levels of all substrates of a given type.

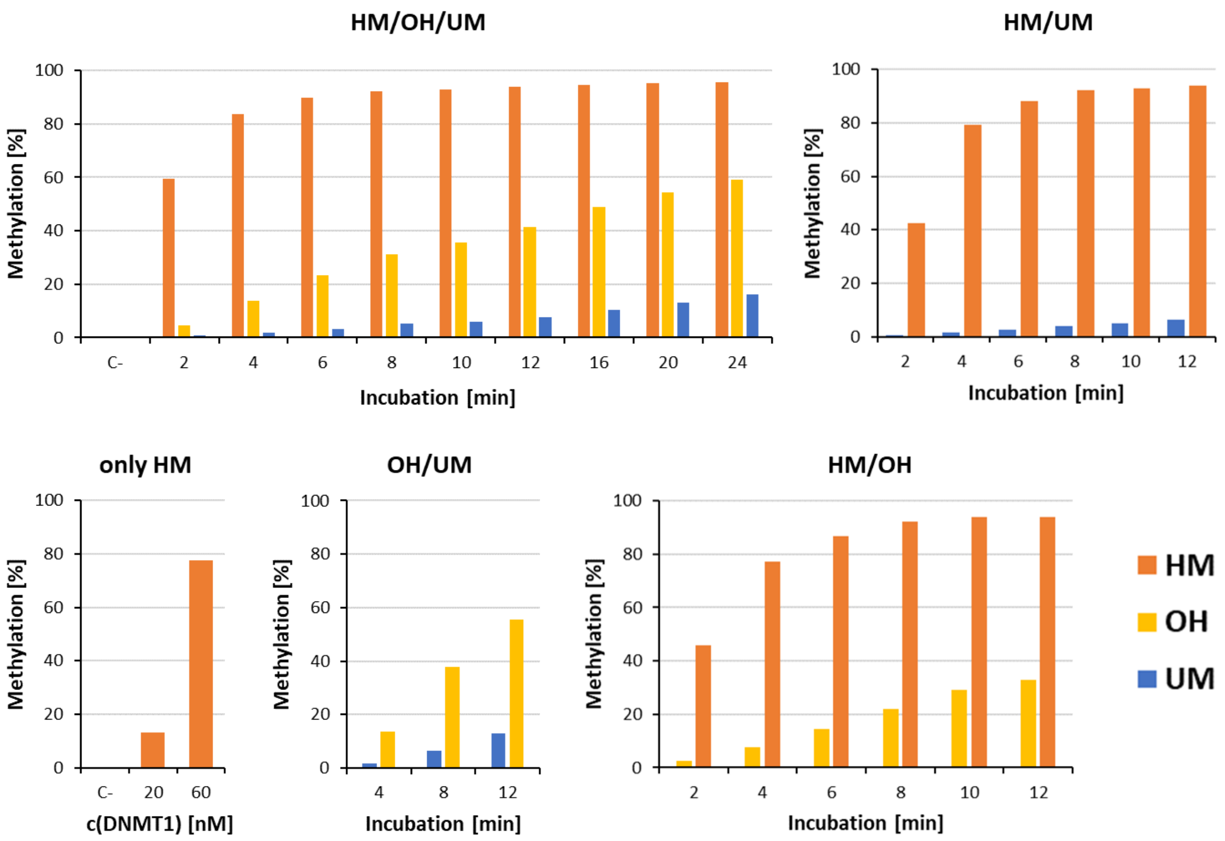

Supplemental Figure 6: Flanking sequence preferences of DNMT1 for the −8 to +8 region on HM, OH and UM substrates. Profiles are based on the observed/expected ratios of nucleotide distribution in the methylated and unmethylated sequence reads. Shown are averages of profiles of individual time points, experiments and repeats and their SEM. N=21 (including two data sets from Adam et al., 2020) in case of HM, 18 in case of OH and UM.

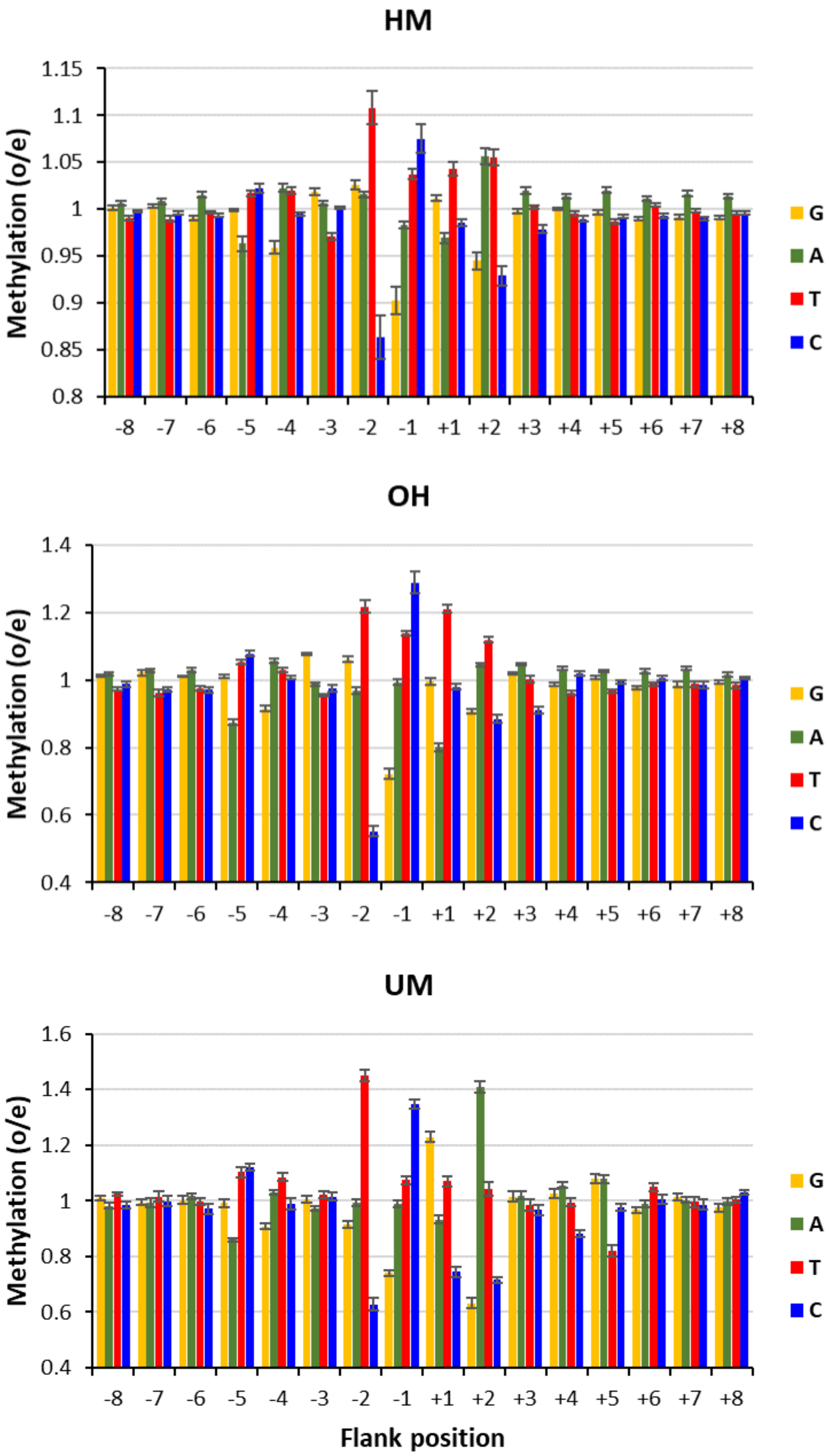

Supplemental Figure 7: Data processing of individual experiments. A) Correlation of NNCGNN methylation rates of HM, OH and UM substrates determined by fitting of the methylation time courses in the individual experimental settings. B) Scaling of experiments to comparable activity levels. C) Distribution of the SEM values of the averaged NNCGNN methylation rates. Boxes show the median, 1st and 3rd quartile. Whiskers display the 1.5 IQR distance. Outliers are indicated by dots. X indicates the average.

A

		HM alone	HM/UM		HM/OH		OH/UM		HM/OH/UM		
			HM	UM	HM	OH	OH	UM	HM	OH	UM
HM alone		1.00	0.89	0.76	0.90	0.88	0.89	0.69	0.91	0.89	0.81
HM/UM	HM	0.89	1.00	0.78	0.92	0.90	0.90	0.77	0.91	0.88	0.82
	UM	0.76	0.78	1.00	0.75	0.70	0.69	0.87	0.74	0.68	0.92
HM/OH	HM	0.90	0.92	0.75	1.00	0.88	0.90	0.71	0.90	0.89	0.79
	OH	0.88	0.90	0.70	0.88	1.00	0.96	0.64	0.90	0.97	0.74
OH/UM	OH	0.89	0.90	0.69	0.88	0.96	1.00	0.63	0.90	0.97	0.73
	UM	0.69	0.77	0.87	0.71	0.96	0.63	1.00	0.70	0.63	0.88
HM/OH/UM	HM	0.91	0.91	0.74	0.90	0.90	0.90	0.70	1.00	0.90	0.78
	OH	0.89	0.88	0.68	0.89	0.97	0.97	0.63	0.90	1.00	0.73
	UM	0.81	0.82	0.92	0.79	0.74	0.73	0.88	0.78	0.73	1.00

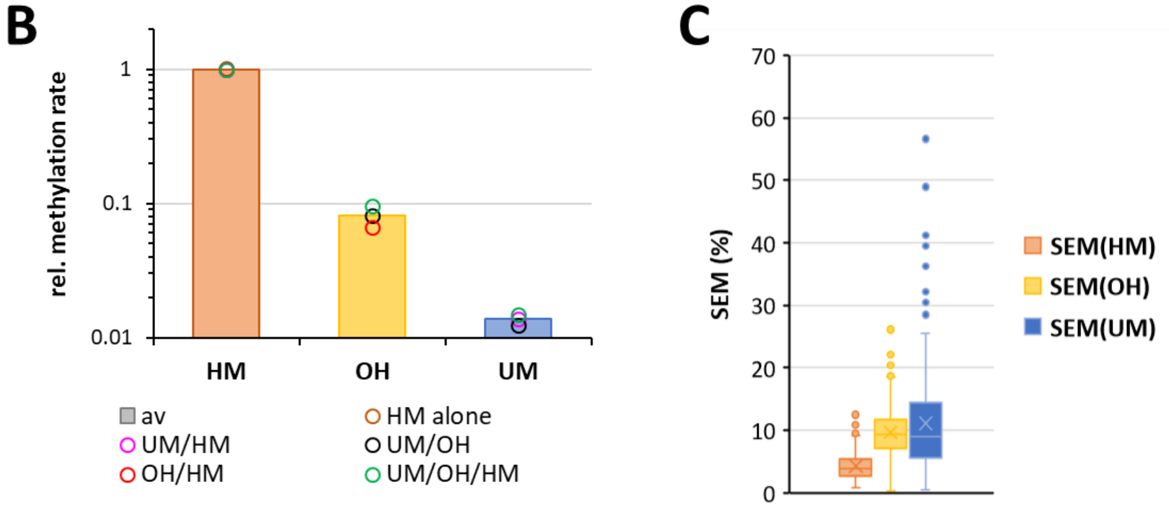

Supplemental Figure 8: Experimental validation of substrate preferences by radioactive DNA methylation kinetics using two model substrates in HM, OH and UM state. The rank of the substrates refers to the NNCGNN methylation rates, a small number indicates a high preference. Substrate sequences are given in Supplemental Table 1.

	Seq.	Rank HM	Rank OH	Rank UM
**Good**	TACGGA	37	52	3
**Bad**	CTCGCG	182	169	214

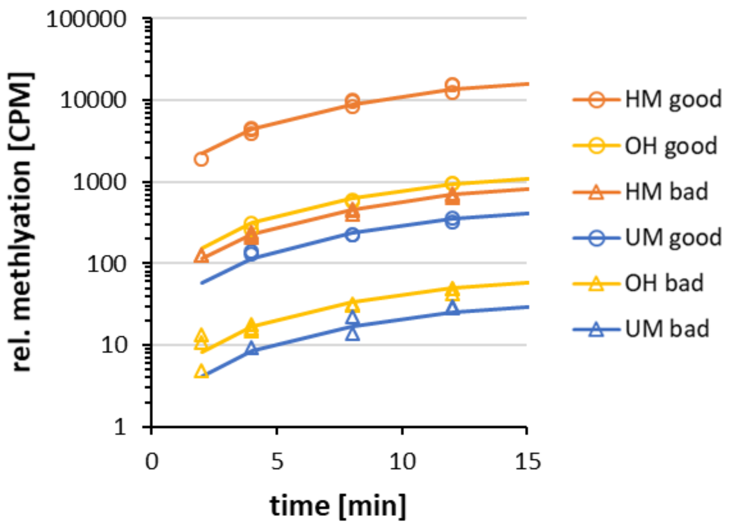

Supplemental Figure 9: Profiles of the DNMT1 methylation reactions with mixed long HM, UM and patterned substrates sorted by reaction progress. The first panel shows the no-enzyme control. Yellow shading highlights the hemimethylated sites on the patterned substrate. UM, completely unmethylated substrate; HM, substrate only containing hemimethylated CpG sites; Patterned substrate is hemimethylated at GCGC and CCGG sites and unmethylated at all other CpG sites.

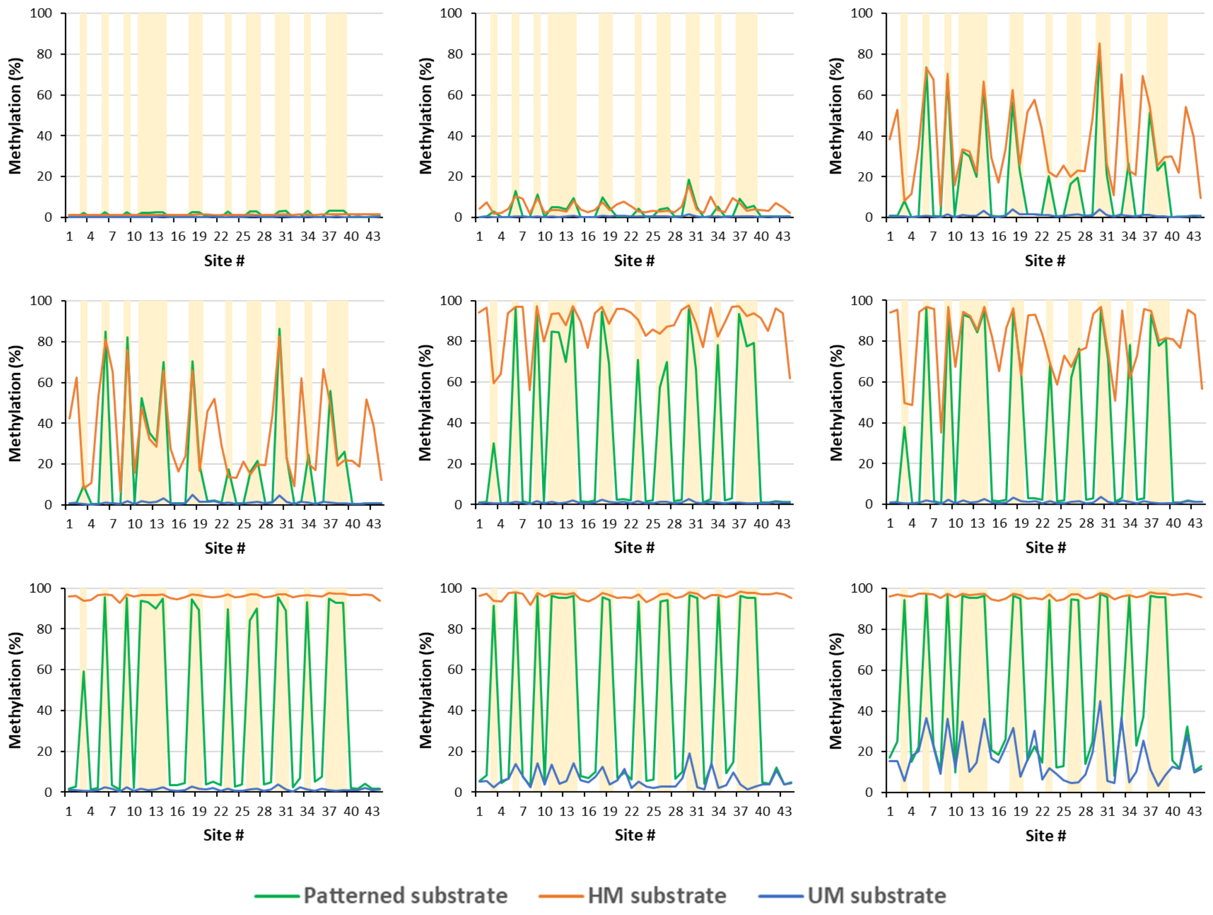

Supplemental Figure 10: Methylation reactions on libraries of mixed HM and UM substrates with randomized -10 to +10 flank carried out with the DNMT1 CXXC mutant. Shown are average methylation levels of the HM and UM sites.

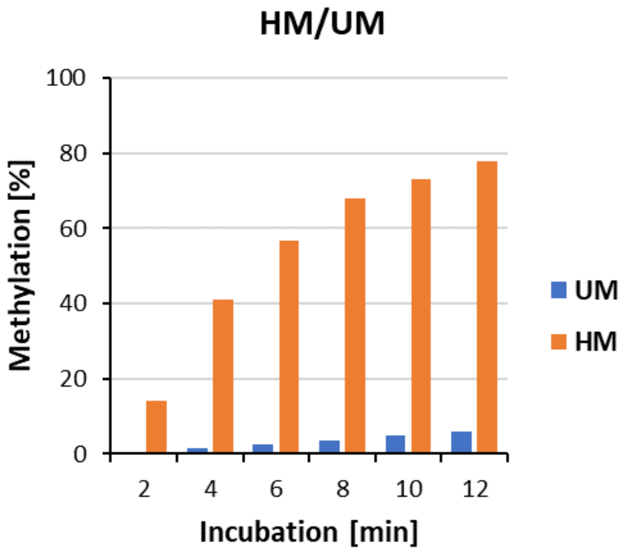

Supplemental Figure 11: Comparison of the methylation rates of WT DNMT1 and CXXC mutant on each NNCGNN substrate (taken from Figure 4B). The substrates used in two studies investigating the effect of the CXXC domain on the HM/UM specificity of DNMT1 are indicated. They either observed an important role of the CXXC domain in the specificity (Song et al., 2011) (1), or did not observe such an effect (Bashtrykov, 2012) (2).

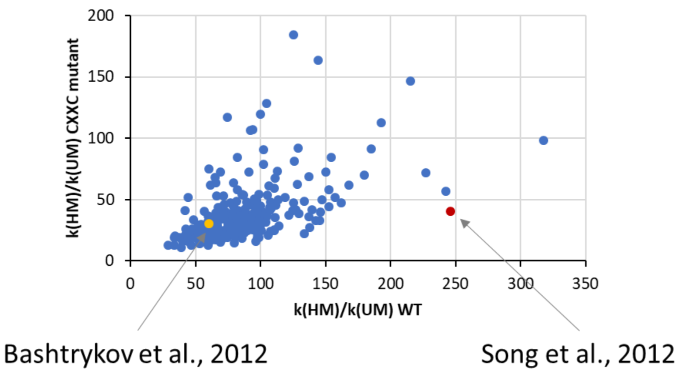

Supplemental Figure 12: Profiles of the CXXC mutant methylation reactions with long patterned substrates. A) Methylation of the long patterned substrate by the CXXC mutant. B) Corresponding WT DNMT1 data were extracted from Supplemental Figure 7. The lines show different time points of the reactions. Yellow shading highlights the hemimethylated sites on the patterned substrate.

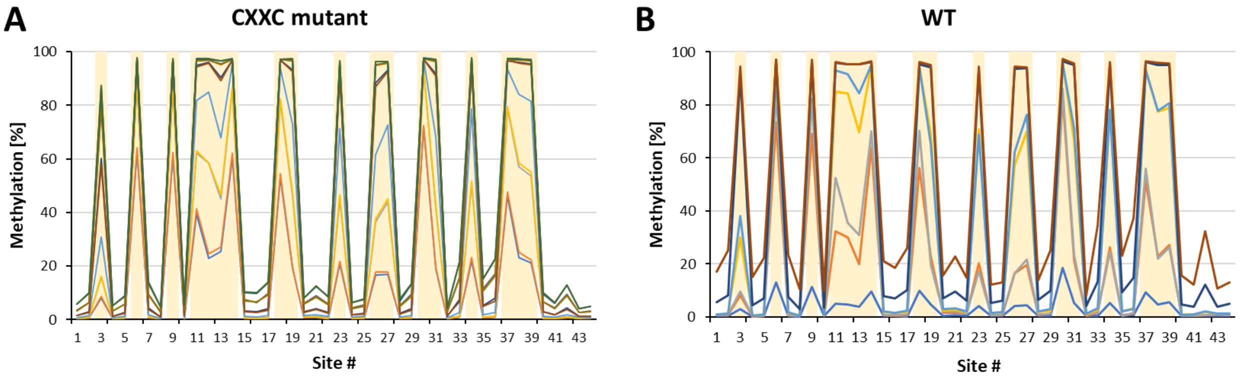

Supplemental Figure S13: Correlation of the flanking sequence preferences of different DNMTs and TET enzymes. Pearson correlation factors of DNMT1 HM and UM flanking sequence preferences determined here with reanalyzed data for DNMT3A (Gao et al. 2020; Dukatz et al. 2022) (3,4), DNMT3B (Dukatz et al. 2020) (5), and TET1 and TET2 5mCG oxidation NNCGNN flanking sequence preferences (Adam et al. 2022) (6).

	DNMT1 HM	DNMT1 UM	DNMT3A	DNMT3B	TET1	TET2
**DNMT1 HM**	1.00	0.82	0.20	0.03	0.20	0.13
**DNMT1 UM**	0.82	1.00	0.24	0.11	0.26	0.18
**DNMT3A**	0.20	0.24	1.00	0.31	0.07	-0.01
**DNMT3B**	0.03	0.11	0.31	1.00	0.32	0.32
**TET1**	0.20	0.26	0.07	0.32	1.00	0.84
**TET2**	0.13	0.18	-0.01	0.32	0.84	1.00

Supplemental Tables

Supplemental Table 1: Oligonucleotides used in the radioactive methylation kinetics.

Good substrate	Upper strand	Bt-TTGCACTCTCCTTCCGTAAGTCCCAGCTTC
	Lower strand UM	GAAGCTGGGACTTACGGAAGGAGAGTGCAA
	Lower strand OH	GAAGCTGGGACTTA^{5hm}CGGAAGGAGAGTGCAA
	Lower strand HM	GAAGCTGGGACTTA^{5m}CGGAAGGAGAGTGCAA
Bad substrate	Upper strand	Bt-TTGCACTCTCCTCGCGAGAGTCCCAGCTTC
	Lower strand UM	GAAGCTGGGACTCTCGCGAGGAGAGTGCAA
	Lower strand OH	GAAGCTGGGACTCT^{5hm}CGCGAGGAGAGTGCAA
	Lower strand HM	GAAGCTGGGACTCT^{5m}CGCGAGGAGAGTGCAA

Supplemental Table 2: Sequencing statistics for the methylation experiments of single CpG site substrates in randomized sequence context.

Experiment	c(DNMT1)	Incubation time [min]	HM substrate			OH substrate			UM substrate		
			met	umet	%met	met	unmet	%met	met	unmet	%met
HM/UM	0.13 µM	2	17575	23799	42.48	-	-	-	202	31063	0.65
		4	24231	6287	79.40	-	-	-	514	28849	1.75
		6	31588	4283	88.06	-	-	-	999	34943	2.78
		8	37896	3163	92.30	-	-	-	1549	36309	4.09
		10	33900	2617	92.83	-	-	-	1765	33090	5.06
		12	32367	2075	93.98	-	-	-	2235	33045	6.34
HM/OH	0.13 µM	2	12031	14279	45.73	946	37345	2.47	-	-	-
		4	9981	2944	77.22	1844	22715	7.51	-	-	-
		6	16322	2503	86.70	4302	25565	14.40	-	-	-
		8	13747	1194	92.01	6694	23575	22.12	-	-	-
		10	13780	917	93.76	5507	13446	29.06	-	-	-
		12	20136	1282	94.01	12054	24784	32.72	-	-	-
OH/UM	0.26 µM	4	-	-	-	5816	37375	13.47	375	22059	1.67
		8	-	-	-	12986	21302	37.87	1501	21133	6.63
		12	-	-	-	24347	19447	55.59	2548	17275	12.85
HM/OH/UM	0.13 µM	2	8306	5663	59.46	1364	29217	4.46	255	35598	0.71
		4	13458	2627	83.67	4769	29723	13.83	718	37716	1.87
		6	14207	1594	89.91	8569	27935	23.47	1281	37753	3.28
		8	13578	1134	92.29	10109	22333	31.16	1691	31020	5.17
		10	17133	1293	92.98	15167	27366	35.66	2611	41567	5.91
		12	18561	1224	93.81	17760	24981	41.55	3645	44804	7.52
		16	17340	974	94.68	21255	22323	48.77	4708	39913	10.55
		20	17384	870	95.23	21293	17866	54.38	5839	39062	13.00
		24	15582	738	95.48	25127	17385	59.11	6206	32421	16.07
HM/OH/UM	No enzyme	-	28	9014	0.31	68	28227	0.24	71	30002	0.24
HM*)	0.062 µM	12	104705	30008	77.72	-	-	-	-	-	-
	0.012 µM	12	18284	124351	12.82	-	-	-	-	-	-

Supplemental Table 3: Sequencing statistics for the methylation experiments with long DNA substrates. UM, completely unmethylated substrate; HM, substrate only containing hemimethylated CpG sites; Patt, substrate is hemimethylated at GCGC and CCGG sites and unmethylated at all other CpG sites.

Substrate	c(DNMT1)	Incubation time [min]	# reads		
			UM	Patt	HM
HM/ Patt/ UM	0.265 µM	1	55463	25045	25727
		3	55983	16897	17841
		10	42584	17389	26144
		30	36643	30874	40261
	1.06 µM	1	50515	26983	26928
		3	33177	12981	10882
		10	87139	27248	39331
		30	107881	32876	44885
	0 µM	0	54814	29485	29594

Supplemental Table 4: Sequencing statistics for the methylation experiments with the DNMT1 containing mutations in the CXXC domain.

Experiment	c(CXXC mutant)	Incubation time [min]	HM substrate			UM substrate		
			met	umet	%met	met	unmet	%met
HM/UM	0.075 µM	2	3579	21778	14.11	103	29618	0.35
		4	9582	13727	41.11	368	26014	1.39
		6	13124	10040	56.66	622	24816	2.45
		8	19835	9346	67.97	1112	29178	3.67
		10	21672	7977	73.10	1534	29626	4.92
		12	18305	5211	77.84	1526	24766	5.80

Substrate	c(CXXC mutant)	Incubation time [min]	replicate	#reads
Patt	0.186 µM	1	1	53301
		3	1	58698
		5	1	60010
		10	1	70105
		20	1	49341
		30	1	55845
		1	2	46588
		3	2	46923
		5	2	37493
		10	2	35733
		20	2	60956
		30	2	60827

Supplemental References

1. Song, J., Rechkoblit, O., Bestor, T.H. and Patel, D.J. (2011) Structure of DNMT1-DNA complex reveals a role for autoinhibition in maintenance DNA methylation. *Science*, **331**, 1036-1040.
2. Bashtrykov, P., Jankevicius, G., Smarandache, A., Jurkowska, R.Z., Ragozin, S. and Jeltsch, A. (2012) Specificity of Dnmt1 for methylation of hemimethylated CpG sites resides in its catalytic domain. *Chemistry & biology*, **19**, 572-578.
3. Gao, L., Emperle, M., Guo, Y., Grimm, S.A., Ren, W., Adam, S., Uryu, H., Zhang, Z.M., Chen, D., Yin, J. *et al.* (2020) Comprehensive structure-function characterization of DNMT3B and DNMT3A reveals distinctive de novo DNA methylation mechanisms. *Nature communications*, **11**, 3355.
4. Dukatz, M., Dittrich, M., Stahl, E., Adam, S., de Mendoza, A., Bashtrykov, P. and Jeltsch, A. (2022) DNA methyltransferase DNMT3A forms interaction networks with the CpG site and flanking sequence elements for efficient methylation. *The Journal of biological chemistry*, **298**, 102462.
5. Dukatz, M., Adam, S., Biswal, M., Song, J., Bashtrykov, P. and Jeltsch, A. (2020) Complex DNA sequence readout mechanisms of the DNMT3B DNA methyltransferase. *Nucleic Acids Res*, **48**, 11495-11509.
6. Adam, S., Bracker, J., Klingel, V., Osteresch, B., Radde, N.E., Brockmeyer, J., Bashtrykov, P. and Jeltsch, A. (2022) Flanking sequences influence the activity of TET1 and TET2 methylcytosine dioxygenases and affect genomic 5hmC patterns. *Commun Biol*, **5**, 92.
